# Isolation and characterization of a subtype C avian metapneumovirus circulating in Muscovy ducks in China

**DOI:** 10.1186/s13567-014-0074-y

**Published:** 2014-07-25

**Authors:** Shikai Sun, Feng Chen, Sheng Cao, Jiajia Liu, Wen Lei, Guangwei Li, Yongfeng Song, Junpeng Lu, Chuang Liu, Jianping Qin, Haiyan Li

**Affiliations:** 1 Guangdong Enterprise Key Laboratory for Animal Health and Environmental Control, Department of Poultry Diseases, WENS Group Academy, Guangdong WENS FoodStuff Group Co. Ltd, Yunfu, 527439, Guangdong, China; 2 College of Animal Science, South China Agricultural University, Guangzhou, 510642, Guangdong, China

## Abstract

Subtype C avian metapneumovirus (aMPV-C), is an important pathogen that can cause egg-drop and acute respiratory diseases in poultry. To date, aMPV-C infection has not been documented in Muscovy ducks in China. Here, we isolated and characterized an aMPV-C, designated S-01, which has caused severe respiratory disease and noticeable egg drop in Muscovy duck flocks in south China since 2010. Electron microscopy showed that the isolate was an enveloped virus exhibiting multiple morphologies with a diameter of 20–500 nm. The S-01 strain was able to produce a typical cytopathic effect (CPE) on Vero cells and cause death in 10- to 11-day-old Muscovy duck embryos. In vivo infection of layer Muscovy ducks with the isolate resulted in typical clinical signs and pathological lesions similar to those seen in the original infected cases. We report the first complete genomic sequence of aMPV-C from Muscovy ducks. A phylogenetic analysis strongly suggested that the S-01 virus belongs to the aMPV-C family, sharing 92.3%-94.3% of nucleotide identity with that of aMPV-C, and was most closely related to the aMPV-C strains isolated from Muscovy ducks in France. The deduced eight main proteins (N, P, M, F, M2, SH, G and L) of the novel isolate shared higher identity with hMPV than with other aMPV (subtypes A, B and D). S-01 could bind a monoclonal antibody against the F protein of hMPV. Together, our results indicate that subtype-C aMPV has been circulating in Muscovy duck flocks in South China, and it is urgent for companies to develop new vaccines to control the spread of the virus in China.

## Introduction

Egg-drop represents great economic loss in the poultry industry. There are a number of pathogens that can cause egg-drops, such as avian influenza virus, egg drop syndrome-76 virus, Newcastle disease virus, duck reovirus, duck virus enteritis, goose and duck parvoviruses [1]. A new Tembusu-related *Flavivirus* named BYD virus, which was recently isolated from Peking ducks in China, was reported to cause a reduction in egg production in ducks [2].

Avian metapneumovirus (aMPV), also known as avian *Pneumoviruses* (APV), belongs to the *Paramyxoviridae* family, the *Pneumovirinae* subfamily, and the *Metapneumovirus* genus. aMPV was first reported in South Africa in 1980 [3], was subsequently reported in France [4] and the United Kingdom [5] and was recently described worldwide [6]–[9]. Based on the antigenicity and genetic characterization, aMPV was further categorized into four subtypes, designated A, B, C and D [10],[11]. aMPV is an enveloped, single-stranded, negative-sense RNA virus. It contains eight genes that encode proteins in the order of 3′-N-P-M-F-M2-SH-G-L-5′ [12]. To date, only one serotype of aMPV has been described. Subtype C aMPV most closely resembles the newly discovered human metapneumovirus (hMPV) in comparison to the other three subtypes [13]. Turkeys and chickens are most commonly susceptible to aMPV infection [12]. Mallard ducks, pheasants, guinea fowl, ostriches and geese can also be infected by aMPV [7],[14]–[16]. Wild birds and seagulls are possible carriers of aMPV, which may explain why the outbreaks of aMPV infections occur mainly during the spring and fall migratory periods [13].

aMPV-C infection in chickens was first reported in China in 2013 [9]. Infection of aMPV-C in Muscovy duck, which has only been seen in France [17], has not yet been documented in China. Since July 2010, a severe Muscovy duck disease with egg-drop, respiratory and ovary-oviduct symptoms has been spreading in the Muscovy duck-producing regions in South China. The causative agent of this disease was unknown at the time. Here, for the first time, we isolated and characterized a subtype C aMPV, S-01, from the affected Muscovy ducks in China, and found that S-01 is the causative agent of this disease. Our findings suggest that subtype C aMPV has been circulating in Chinese poultry and effective strategies should be taken immediately to prevent the spread of the virus.

## Materials and methods

### Ethics statement

The animal slaughter experiments were conducted in accordance with the guidelines of the Guangdong Province on the Review of Welfare and Ethics of Laboratory Animals approved by the Guangdong Province Administration Office of Laboratory Animals (GPAOLA). All the animal procedures were conducted under the protocol (SCAU-AEC-2010-0416) approved by the Institutional Animal Care and Use Committee (IACUC) of South China Agricultural University.

### Sample preparation

Clinical samples were collected from infected ducks (the duck owners provided consent for all slaughter experiments for privately owned ducks) from 2010 to 2012. A total of 60 duck flocks (50 layer and 10 breeder flocks) with clinical signs were examined. Nasopharyngeal swabs, cloacal swabs, ovary, uterus, larynx, trachea and nasal turbinate specimens were collected. The nasopharynx and cloacae of eighteen birds were swabbed per flock. The nasopharyngeal swabs or cloacae swabs were pooled and suspended in minimum essential medium with penicillin-streptomycin, followed by centrifugation at 12 000 × *g* for 1 min at 4 °C. The supernatant immediately underwent RNA extraction and virus isolation. The ovary, uterus, larynx, trachea and nasal turbinates were cut into pieces and suspended with a mixture of Phosphate Buffered Saline (PBS) containing penicillin-streptomycin. The suspension was centrifuged at 12 000 × *g* for 1 min at 4 °C, and the supernatant was used immediately for RNA extraction and virus isolation.

Statistical analysis was performed on daily egg production rate represented by 6 different flocks of diseased Muscovy ducks from three different farms. Daily egg production rate of two normal flocks from another farm was analyzed as the control. The birds in these four farms were 38 (Farm 1-F1), 35 (Farm 1-F2), 43 (Farm 2-F1), 45 (Farm 2-F2), 45 (Farm 3-F1), 36 (Farm 3-F2), 35 (Farm 4-F1) and 43 (Farm 4-F2) weeks old, respectively.

### Viral RNA extraction, PCR and sequence analysis

Total RNA was extracted directly from the treated samples using the RNeasy kit (AxyPrep, Union City, USA) according to the manufacturer’s instructions. The TaKaRa PrimeScript one-step RT-PCR Kit Ver.2 (TaKaRa, Dalian, China) was used for RT-PCR. All PCR reactions were carried out using the PCR machine (Biometra, Goettingen, Germany). aMPV-C was detected by RT-PCR as described by Ali and Reynolds [18]; BYD virus was detected by RT-PCR as described by Yan et al. [19] and Su et al. [2]; avian influenza virus was detected by RT-PCR as described by Fereidouni et al. [20]; Newcastle disease virus was detected by RT-PCR as described by Gohm et al. [21]; duck reovirus was detected by PCR as described by Zhang et al. [22]; goose parvovirus was detected by PCR as described by Limn et al. [23]; Egg drop syndrome-76 virus was detected by PCR as described by Kumar et al. [24]; duck parvovirus was detected by PCR as described by Sirivan et al. [25].

aMPV was purified by 5 passages on Vero cells, and the aMPV structural genes were amplified by RT-PCR using specific primers, as shown in Table 1. The RT-PCR reactions were performed using 2 μL of PrimeScript 1 Step Enzyme Mix, with final concentrations of 2 × 1 Step Buffer (25 μL), 11 μL of RNase free H_2_O, 1 μL of each primer in a total reaction volume of 50 μL containing 10 μL of RNA. The reaction mixtures were subjected to amplification with the following temperature conditions: 50 °C for 30 min for reverse transcription (RT), 95 °C for 5 min for initial denaturation, followed by 30 cycles of denaturation at 94 °C for 1 min, annealing at 55 °C for 1 min, and extension at 72 °C for 2 min. The final extension was carried out at 72 °C for 10 min. We designed 9 pairs of overlapping primers (Table 1) according to the complete genome sequence of subgroup C aMPV Colorado strain (GenBank accession number: AY590688). Nine pairs of primers divided the full genome of the new isolate into nine overlapping segments ranging from 800 to 2300 base pairs (bp). The overlapping regions that used to ensure the accuracy of sequencing ranged from 15 to 150 bp. Virus genomic cDNA sequence excluding the 5′- and 3′- terminus was eventually determined. All sequences from independent RT-PCR products were sequenced 3–4 times in both directions and are reported in the present paper as cDNA.

**Table 1:** **Primers used in this study**^**a**^

**Name**	**Sequence (5′-3′)**	**Position**	**Target gene**
N-F	5′ GGGACAAGTGAAAATGTCTCTTCAGGGGATTCA GCTTA 3′	41-77	N
N-R	5′ TTTTTAATTACTCATAATCATTCTGGCCT 3′	1235-1263	
P-F	5′ GGGACAAGTCAAAATGTCCTTTCC 3′	1248-1271	P
P-R	5′ GTTTTTTATTAACTACATAGTAAGGGAGTATAG GTCATC 3′	2136-2174	
M-F	5′ GGGGACAAGTIAAIATGGAGTC 3′	2157-2175	M
M-R	5′ GTCTTGGCTATCGCTACACC3′	3399-3418	
F-F	5′ GGGACAAGTGAAAATGTCTTGG 3′	3208-3229	F
F-R	5′ TCTTCACTTGTCCCAATTTTTT 3′	4666-4687	
M2SH-F	5′ TTGGGACAAGTGAAGATGTCTCG 3′	4672-4694	M2-SH
M2SH-R	5′ CTTGACTTTGACTTTAAGCTCCTG 3′	6186-6209	
L1-F	5′ GGACCAAGTTAAAAATGGATCCAC 3′	7939-7962	L
L1-R	5′ TACACTCCTTCTGTTTCTGGAGG 3′	10006-10028	
L2-F	5′ TGGCTGCATTTGACTGTTCCACT 3′	9951-9983	L
L2-R	5′ CACTAAGTCCAATTTGTCAGGG 3′	12068-12089	
L3-F	5′ TACAGGCAGAAGCCCTAAACAAT 3′	11933-11955	L
L3-R	5′ GCAAAAAAACCGTATTCATCC 3′	14126-14146	
G-F	5′ CAGCCAGGCAATCACACAACAGTTC 3′	5881-5904	G
G-R	5′ CCACTTTCGAAGTGTTATCCTTTTT 3′	8076-8099	

^a^Primers according to available subgroup C aMPV Colorado strain, GenBank accession number: AY590688.

### Sequence analysis

After sequencing fragments, nucleotide sequence editing and prediction of aa sequences were accomplished using the DNAStar (Madison, WI, USA). Multiple-sequence alignments were generated with ClustalX 2.0, and nucleotide sequence homologies were further obtained through the Clustal W method of DNAStar [26]. All sequencing data were assembled using DNAStar. Phylogenetic trees were generated by the neighbor-joining tree method using MEGA 5.1 software, and bootstrap values were calculated on 1000 replicates of the alignment [27]. Every ORF and most of the deduced amino acids were compared with reference isolates.

### Isolation of aMPV

The aMPV-positive samples were inoculated in 11-day-old duck embryos via the yolk sac. The yolk fluid was collected 6 days post-inoculation. The yolk fluid collected from the inoculated embryos was further examined for the presence of aMPV by RT-PCR as described by Ali and Reynolds [18]. The embryo egg yolk and allantoic fluid from the duck embryos were mixed thoroughly and used for inoculation of Vero cells until the embryo’s death was evident. When CPE was observed, a TCID_50_ assay was conducted to determine the titer of the virus as described by Brian et al. [28].

### Electron microscopic analysis

Virus-infected cells were harvested and concentrated via ultracentrifugation at 40 000 × *g* for 1 h at 4 °C. The virion particles in the pellet were visualized by electron microscopy (EM) as described by Kwon et al. [29].

### Indirect immunofluorescence assay (IFA)

Vero cells in 24-well plates (1 × 10^5^ cells/well) were infected with the isolated virus (10^5^ TCID_50_) for 48 h. The cells were then fixed with 100% cold methanol for 10 min at 4 °C. The fixed cells were then incubated with a monoclonal antibody (MAB80123, Temecula, MILLIPORE) against the F protein of hMPV as described by Luo et al. [10].

### Growth kinetics

Vero cells in 6-well plates were infected with aMPV at an MOI of 0.1. After incubation at 37 °C for 1 h, the inoculates were removed. The cells were washed twice with 1× PBS and incubated with DMEM containing 10% fetal bovine serum (FBS) at 37 °C. At the designated time points, the cell culture supernatants were harvested and titrated by a standard TCID_50_ assay.

### Serological study

Sera were collected from convalescent laying Muscovy ducks and control birds. Sera were tested for the presence of aMPV antibodies using the Avian Pneumovirus Antibody Test Kit (IDEXX, Liebefeld-Bern, Switzerland) according to the manufacturer’s instructions. In order to make the kit suitable for detection of duck immunoglobulins, we replaced the Anti-Chicken HPRO (which was provided with the kit) with Anti-Duck HPRO conjugate for the ELISA test. Based on the optical density (*OD*) values at 405 nm, sample to positive (S/P) ratios were calculated, and the average S/P ratio was used to evaluate the level of antibodies in each group. Serum samples with S/P ratios greater than 0.2 (titers greater than 396) were considered to possibly contain anti-aMPV antibodies, and a ratio of 0.2 or lower (titers less than or equal to 396) were considered negative, based on the manufacturer’s recommendations.

### Neutralization test

To rule out the presence of other viruses in the S-01-containing suspension, sera of anti-reovirus, −avian influenza virus (H5N1 and H9N2), −Newcastle disease virus, −goose parvovirus, −avian pneumovirus, and -BYD virus were used to interact with the S-01 virus at 4 °C for 3 h. The virus-antibody mixtures were transferred into Vero cells in 24-well plates and incubated at 37 °C. When CPE was evident, the cells infected or mock-infected with S-01 or S-01/ anti-serum were detected by IFA, as described by Luo et al. [10].

### In vivo experiments

To evaluate the pathogenicity of the S-01 virus, thirty-five 20-day-old breeding Muscovy ducks, and 200 300-day-old laying Muscovy ducks were obtained from Rencun and Zhejiang Subsidiaries of Guangdong WENS Foodstuff Group Co. Ltd (Yunfu, China and Changshan, China, respectively). All ducks were housed and bred in a negative pressure and pathogen-free Animal Isolation Facility at room temperature (20 ~ 25 °C), relative humidity of 60%, subjected to a 16 h-light per day with average of 20 ~ 35 lux, supplied with water and feed *ad libitum*. After acclimatization to the new environment for 7 days to minimize the effects of shipping stress, the ducks were carried out for two series of experiments.

The 200 laying Muscovy ducks were randomly divided into five groups (40 ducks/group). The ducks in groups 1, 2 and 3 were infected with F1-, F5-, F10-embryo-passaged S-01 virus, respectively, and every laying duck was infected with S-01 by intranasal (0.5 × 10^5^ EID_50_) and cloacal (10^5^ EID_50_) injections. The ducks in group 4 were mock-infected with 1.5 mL of sterile DMEM containing 4% FBS in the same manner as the control. Ducks in group 5 did not receive any treatment as the negative control. The 35 breeding ducks were divided into four groups. Ducks in groups 1 and 2 were infected respectively with 10^5^ EID_50_ of F5-embryo-passaged and 10^5^ TCID_50_ of F5-cell-passaged S-01 virus by intranasal injection. Ducks in group 3 were mock-infected with 1.5 mL of sterile DMEM containing 4% FBS in the same manner as the control. Group 4 had 5 ducks without any treatment as the negative control. The laying ducks were monitored daily for egg production and clinical symptoms, while breeding ducks were monitored only for clinical signs (e.g. respiratory symptoms). On day 7 post-infection, eighteen ducks with typical respiratory symptoms from six inoculated afore-mentioned groups (3 ducks/group) were killed. Meanwhile, six ducks from three control groups (2 ducks/group) were killed. Nasopharyngeal swab & cloacal swab, ovary & uteru, larynx & trachea, nasal turbinates from each duck were collected. Totally 90 tissue samples were collected from 9 experimental groups for pathological examination and virus isolation. Duck embryos and Vero cells were used to recover the virus from tissue samples. The recovered virus was subsequently confirmed by RT-PCR and sequencing.

### Statistical analysis

The data obtained were analyzed by one-way analysis of variance (ANOVA), and the differences between means were compared by the Duncan’s multiple range test (DMRT) using SPSS 17 (SPSS Inc., Chicago, IL, USA). The data, including aMPV-C antibody titer and daily egg production between naturally infected and inoculated laying Muscovy ducks, was analyzed by the Student’s *t*-test. *P* < 0.05 was considered as statistically significant.

## Results

### New disease in Muscovy ducks was strongly related to aMPV infection

**Figure 1:**
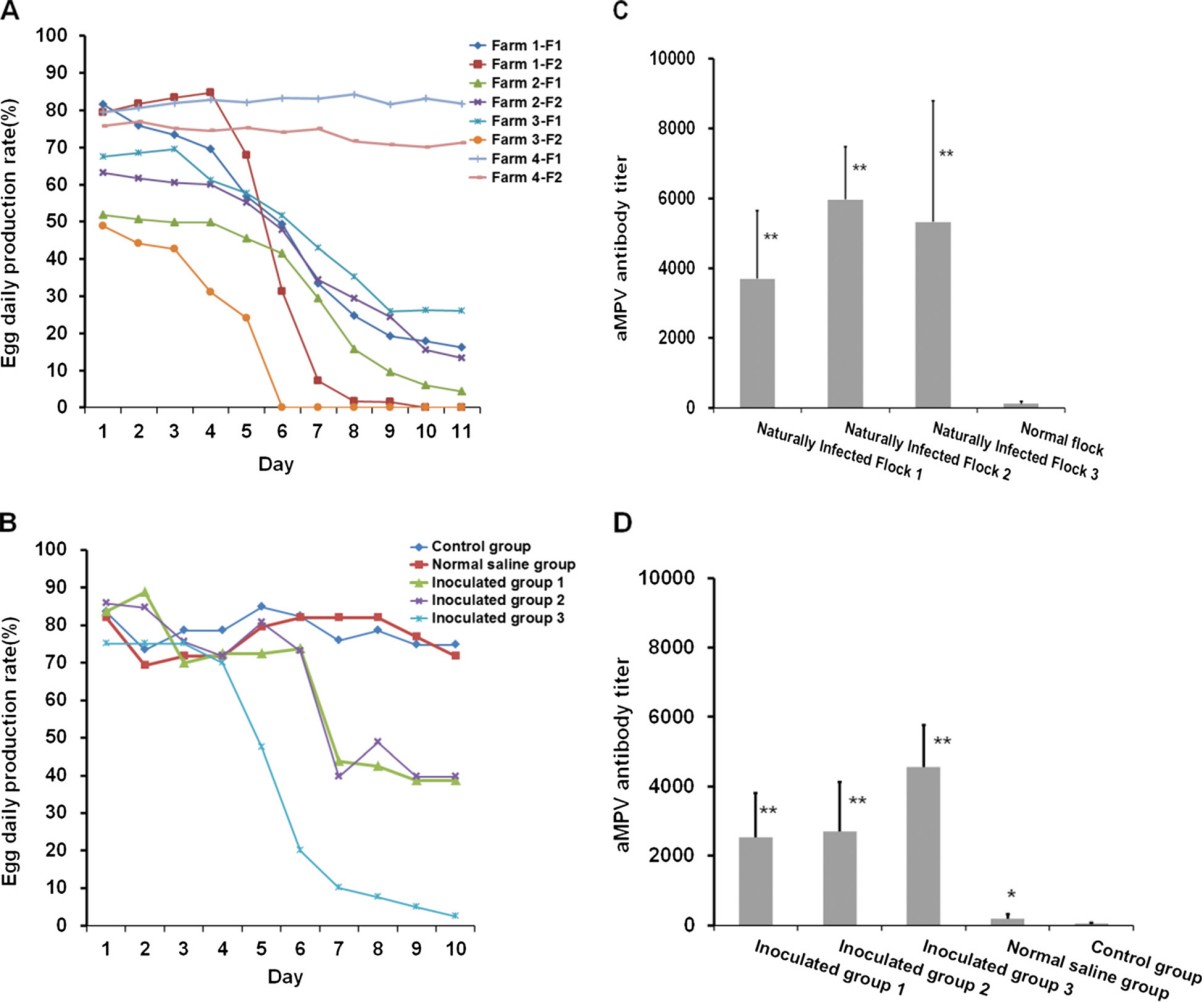
**Daily egg production rate and the aMPV antibody levels of naturally infected and inoculated ducks.** Daily egg production rate represented by different flocks from three infected duck farms (**A**-Farm 1-F1&F2, Farm 2-F1&F2, Farm 3-F1&F2), two normal flocks from Farm 4 were used as the control (**A**-Farm 4-F1&F2). aMPV-antibody titer represented by three groups of ducks naturally infected and recovered (**B** - Naturally infected Flock 1, 2, 3), ducks in the normal flocks were used as the control (**B** - Normal Flock). S-01 was used to reproduce the disease. Ducks in group 1, 2 and 3 were inoculated with F1-, F5-, F10-embryo-passaged S-01 virus, respectively. Daily egg production rate **(C)** and aMPV antibody titer **(D)** are represented by three infected-duck groups and two control groups (Normal saline group, Control group). Statistical significance for an effect upon aMPV-C infection was determined using the Student’s *t*-test. Asterisks indicate statistical differences between control and aMPV-C groups (**P < 0.01; *P > 0.05).

**Figure 2:**
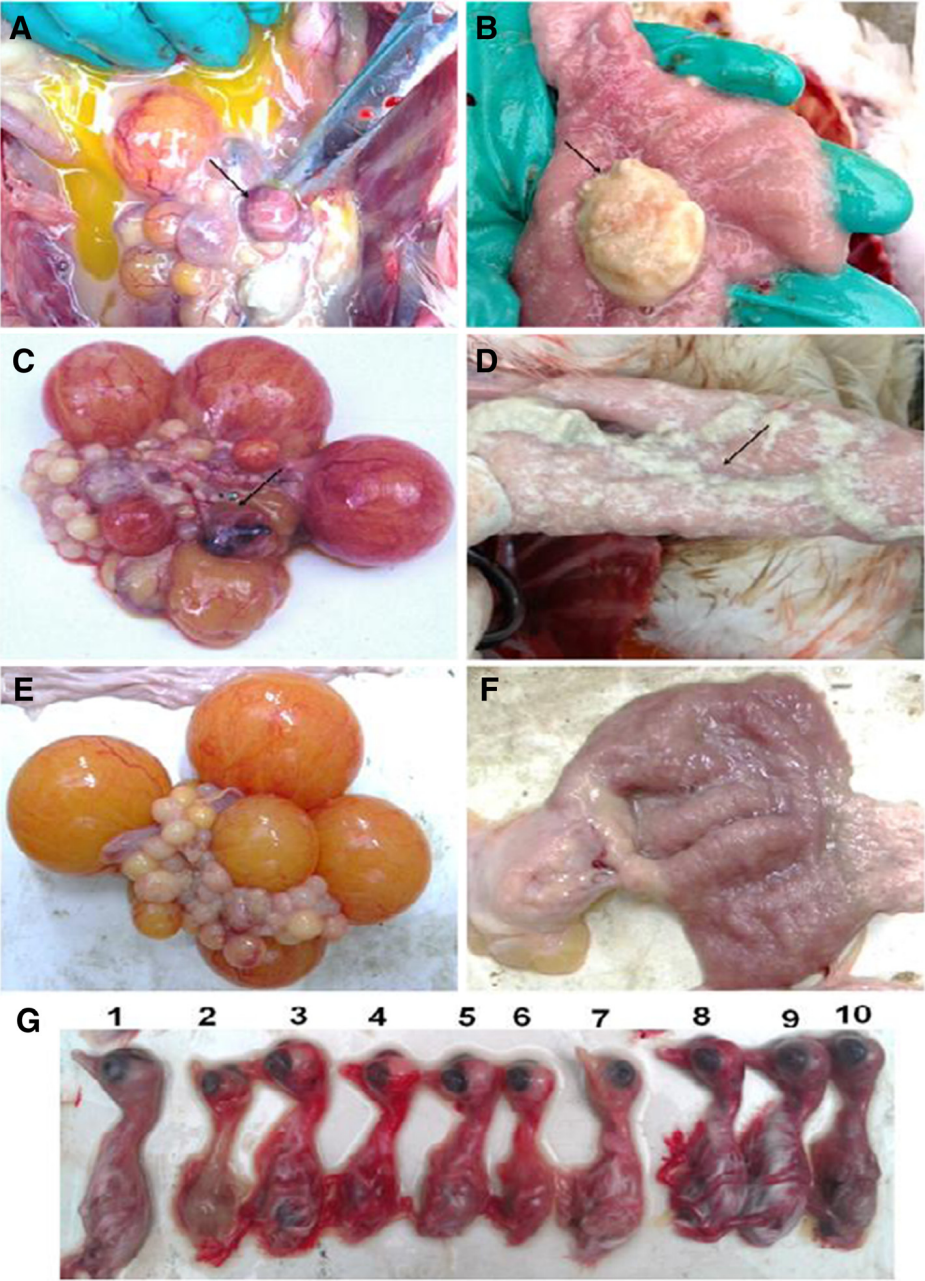
**Pathological studies of the infected ducks and the inoculated duck embryos.** i. The naturally infected ducks were euthanized and anatomic investigations show the prevalence of white or yellow discharge in uterine **(B)**, ovarian hemorrhage and abdominal egg yolk accumulated **(A)**, and uterine flushing **(A)**. The artificial infected laying ducks experiencing necropsies revealed white discharge in the uterine **(D)** and ovarian hemorrhage **(C)**. Mock duck uterine **(F)** and ovarian **(E)**. ii. Duck embryos, 5–7 days post inoculation, growth retardation appeared (**G**-2, −4, −5, −6), haemorrhage (**G**-3, −4, −5, −6, −8, −9, −10), and death (**G**-2, −3, −4, −5, −6, −7, −8, −9, −10), Control (**G**-1).

From July 2010 to July 2012, four farms consisting of 50 flocks of egg-laying Muscovy ducks and 10 flocks of breeding Muscovy ducks were monitored and investigated. An outbreak in Muscovy ducks, with severe symptoms of coughing and egg-drop, occurred in these flocks in Guangdong, China. The outbreak occurred in a seasonal pattern, mainly in early spring and late autumn. Only Muscovy ducks were infected, indicating that the pathogen was species-specific. We observed upper respiratory symptoms and reduction of egg production by approximately 40-85% in the sick Muscovy ducks (Figure 1A, Farm 1, Farm 2 and Farm 3), compared with that of normal Muscovy ducks (Figure 1A, Farm 4). The eggs produced by the diseased ducks were soft, thin-shelled, or cracked (data not shown). In most cases, the symptoms lasted approximately 9–12 days and disappeared rapidly. A secondary infection often occurred afterwards. Anatomical studies showed a prevalence of white or yellow discharge in the uterine (Figure 2B) and nasal tissues (data not show). Lesions and an accumulation of egg yolk were observed in the ovarian tissues (Figure 2A).

To determine whether any of these viruses, including BYD virus, avian influenza virus, Newcastle disease virus, duck reovirus, goose parvovirus, Egg drop syndrome-76 virus and duck parvovirus, were related to the decreased egg production in Muscovy ducks, PCR was initially performed to examine the presence of these viruses in the ovary, uterus, larynx, trachea, nasal turbinates and egg yolk of the ducks. None of the above viruses was detected in PCR (data not shown). Interestingly, the aMPV-C M gene was detected by RT-PCR using total RNA extracted from nasopharyngeal swabs, cloacal swabs, ovary, uterus, larynx, trachea and nasal turbinates of the diseased ducks as a template (data not shown). Using ELISA, we detected the presence of an aMPV-specific antibody in the convalescent sera. The *OD*_450_ values in the sera from the recovered ducks (*n* = 15) (Figure 1B, Naturally Infected Flock 1, 2 and 3) were 50 to 1000-fold higher than that from the control group (*n* = 15) (Figure 1B, Normal Flock). Collectively, our results strongly suggest that the Muscovy ducks were infected with aMPV.

### Novel virus is isolated and characterized as aMPV-like virus

**Figure 3:**
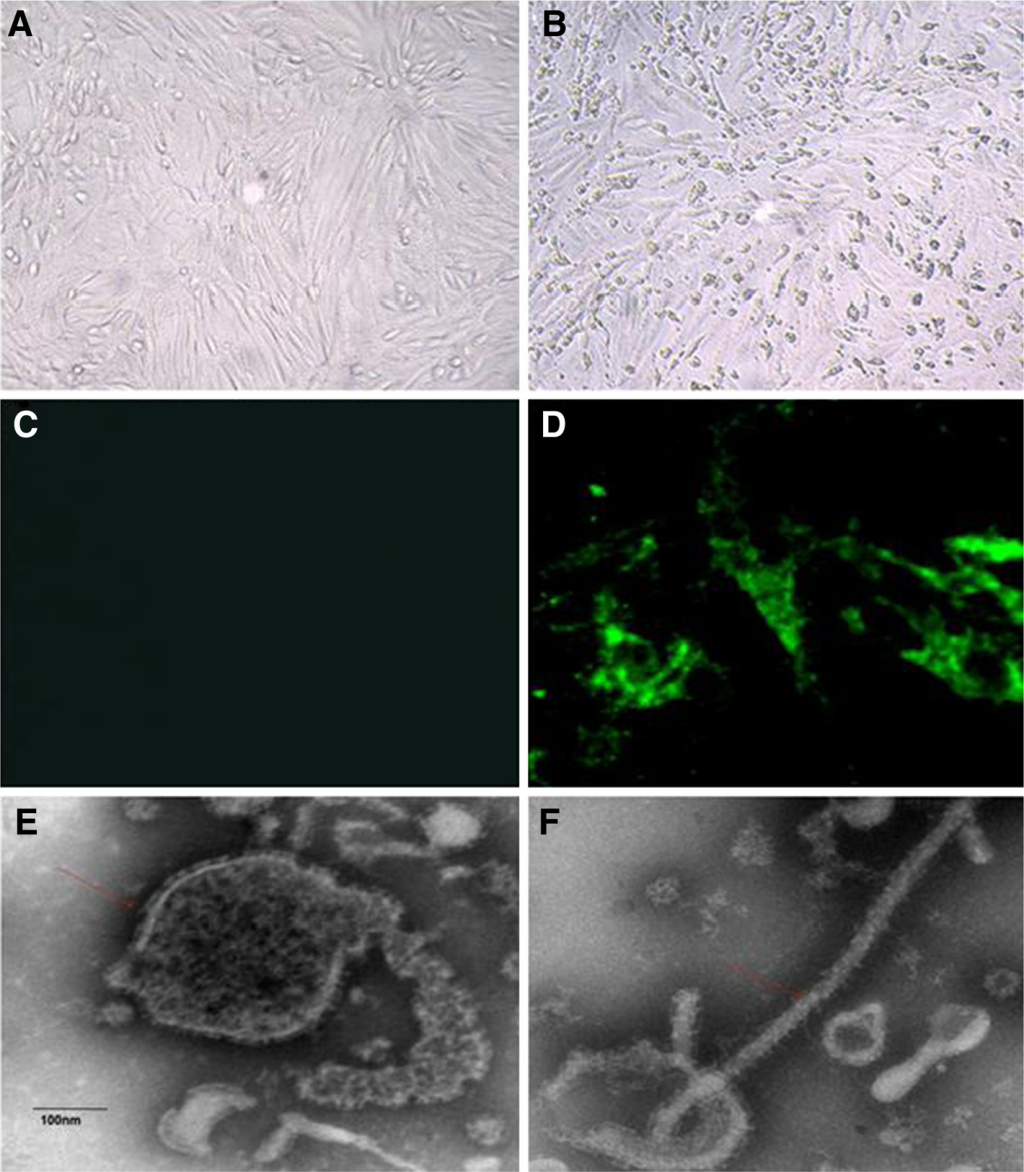
**The CPE observed in infected cells and identification of S-01 by IFA and EM.** i. The Normal Vero cell **(A)** and Vero cell infected with S-01appeared to have an obvious cytopathic effect **(B)**. ii. S-01 could be effectively bound to anti-F protein of human metapneumovirus antibody in Vero cell **(D)**; Mock well with no-infected Vero cell **(C)**. iii. Viral particles observed in Electron micrograph **(E and F)**.

To further verify aMPV as the infective agent, 42 aMPV-positive supernatants collected from clinical samples were inoculated into 11-day-old duck embryos via the yolk sac route. After the third passage, 9 embryos exhibited growth retardation (Figure 2G, embryo No. G-2, −4, −5, −6), bleeding (Figure 2G, embryo No. G-3, −4, −5, −6, −8, −9, −10), and/or death (Figure 2G, embryo No. G-2, −3, −4, −5, −6, −7, −8, −9, −10) at 5–7 days post inoculation, while the control embryos remained normal (Figure 2G, embryo No. G-1). The embryo egg yolks and allantoic fluid collected from the dead embryos were harvested, mixed and used for passing into Vero cells. CPE was observed in the cells infected with virus-containing fluids after the fourth passage at 72 h post inoculation (Figure 3B), and no CPE was observed in the mock-infected cells (Figure 3A).

The isolate was further confirmed by an immunofluorescence assay. There was no antibody available for the subgroup C aMPV, but our amino acid sequencing revealed that the F protein of S-01 was identical to that of aMPV-C and hMPV by 98.5-99.1% and 81.6-81.8%, respectively. Because Luo et al. used recombinant fusion protein [10] to confirm that there was high antigenic cross-reactivity between aMPV-C and hMPV, we used an antibody against the F protein of hMPV for the immunofluorescence assay. The results show that the antigen of S-01 could effectively bind to the antibody in Vero cells (Figure 3D), whereas no fluorescence was observed in mock-infected Vero cells (Figure 3C). The pathogen was serially cultured through 13 passages in Vero cells and then identified by electron microscopy. We observed that the pathogen particle was an enveloped virus characterized by a diameter of 20–500 nm and multiple morphologies (Figure 3E, F), similar to that of aMPV [14],[29]. Vero cells were inoculated with S-01 at an MOI of 0.1. The TCID_50_ values at 24, 48, 72, 96, and 120 hours post-infection (hpi) were 10^2.83±0.30^, 10^4±0.51^, 10^6.47±0.45^, 10^4.75±0.33^ and 10^4.6±0.42^ TCID_50_/0.1 mL, respectively.

A sera neutralization test was used to further analyze whether there were other viruses in the isolate. When the Vero cells were inoculated with the viruses that interacted with the antisera to duck reovirus, avian influenza virus (H5N1 and H9N2), Newcastle disease virus, goose parvovirus, and BYD virus, CPE occurred in the cells at day 3 post-inoculation (data not shown). CPE was also observed in the cells inoculated with the S-01 virus. When the Vero cells were infected with the S-01 virus in the presence of the corresponding antibody, the cells grew as well as the control cells (without infection) (data not shown). In conjunction with the sera neutralization test, the virus suspension was tested by RT-PCR and PCR for miscellaneous potential viruses, such as the duck reovirus, avian influenza virus (H5N1 and H9N2), Newcastle disease virus, goose parvovirus, and BYD virus. We found that only aMPV-C-was detectable (data not shown). Taken together, our findings revealed that the novel isolate, which was able to bind to the monoclonal antibody to F protein of the hMPV and produce a typical CPE in Vero cells, was an aMPV-like enveloped virus, and the best viral replication phase was at approximately 72 hpi.

### Reproduction of the disease with experimental infection

To confirm the identity of the etiological agent of the outbreak, it was essential to reproduce the disease by experimental infections of breeding and laying ducks with the S-01-virus strain. Initially, the infected laying ducks only exhibited symptoms of coughing, which disappeared in 6–7 days, and only a slight egg-drop was observed in the first three reproductions. We adjusted cell-passage strain to the duck embryo-passage strain. The laying duck groups that were infected with embryo-passaged strain of S-01 experienced serious upper respiratory symptoms and egg drop (Figure 1C-Inoculated group 1, 2, 3, the highest drop to approximately 72.5%) at 9 days post-infection, and necropsies revealed similar regression (Figure 2C and D). At 5 days post-infection, all the breeding ducks infected with S-01 displayed severe upper respiratory symptoms, and 3 ducks infected with the F5-embryo -passage strain of S-01 died of secondary infections a week later. The S-01 virus was successfully recovered from the infected ducks and confirmed by RT-PCR, ELISA assay (Figure 1D) and genomic sequencing in subsequent experiments (data not shown).

### Phylogenetic analysis confirms that S-01 belongs to the aMPV-C family

We used a pair of primers specific for the M gene of subgroup C aMPV to detect aMPV-C. The PCR amplification produced a 631-bp fragment, which was consistent with previously published data [18]. Sequence analysis revealed that the amplified fragment shared a 94.9-96.5% nucleotide identity with its corresponding region of the M gene of subgroup C aMPV. When compared to the same region of the M gene of subgroup A or B of aMPV, the amplified fragment shared a nucleotide identity of 70.4-71.8% and 68.9-71.5%, respectively.

**Figure 4:**
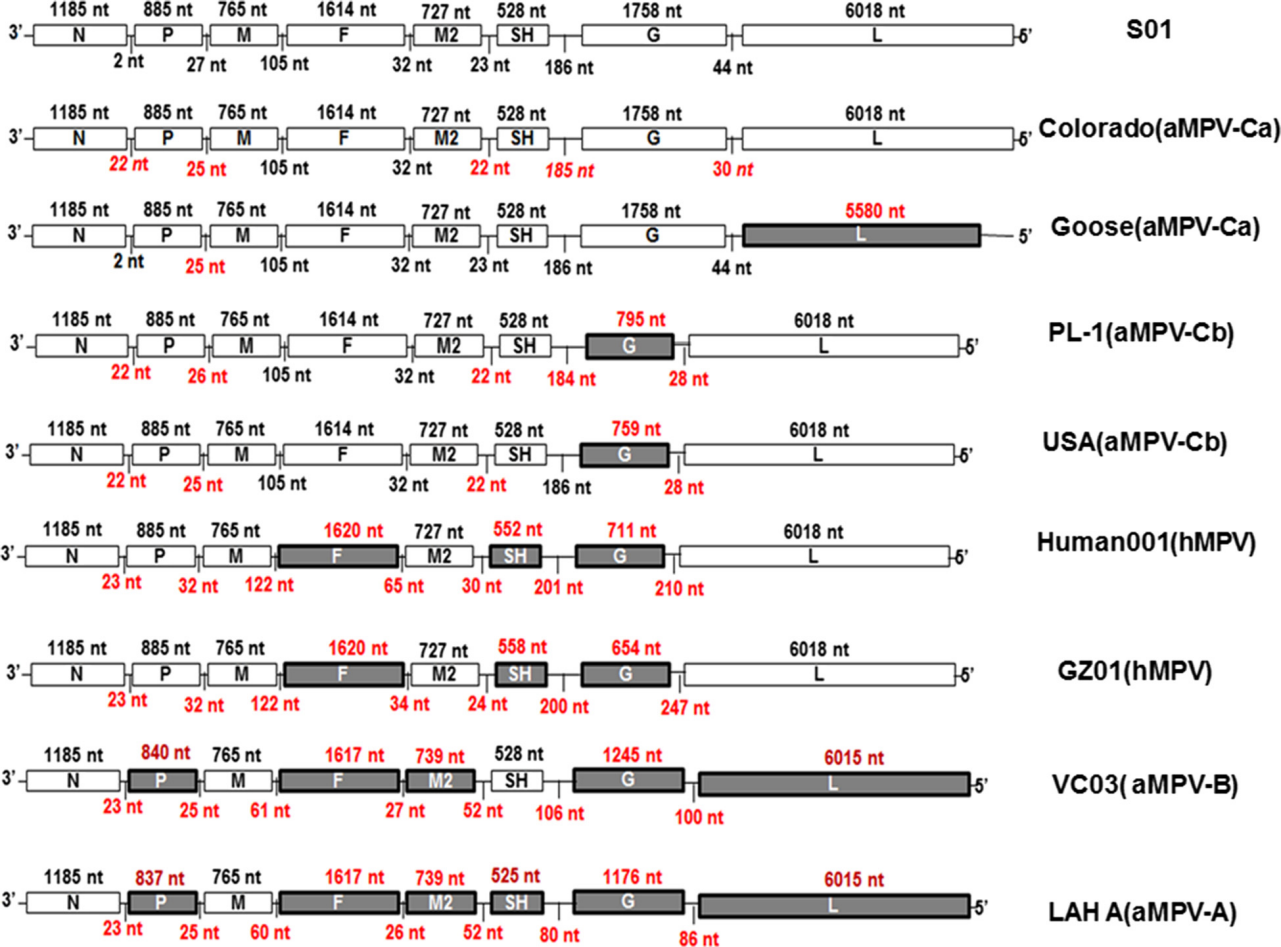
**Viral gene sequence length comparison of completely sequenced aMPV and hMPV.** A comparison of S-01, Colorado (aMPV-C^a^), AY590688, Goose(aMPV-C^a^)/DQ009484, PL-1(aMPV-C^b^)/EF199771, USA (aMPV-C^b^)/AY579780, Human001(hMPV) /AF371337, GZ01(hMPV)/GQ153651, VC03 (aMPV-B)/AB548428 and LAH A (aMPV-A/AY640317 was accomplished. Variation in size of the G, L, SH, N, F and M2 genes was indicated. The length of intergenic regions between the major functional genes were included; nt, Nucleotides. The different regions between the genomic structures are shown in red marks and shaded areas.

After assembling the sequences of all the RT-PCR products, we obtained the entire coding sequence of S-01. The results from a homologous analysis of the genomic sequence and the amino acid sequence indicate that the S-01 virus contained a genomic sequence consisting of 14 079 base pairs (GenBank accession number: KF364615). The sequence of S-01 shared 92.3-94.3%, 69.6-70.4%, 60.4%, 60.2%, 46.6%, 46.2%, 46.1% and 46.4% nucleotide identities with that of aMPV-C, hMPV, aMPV-A, aMPV-B, RSV, BRSV, MPV and HRSV, respectively (Table 2). The nucleotide identities with the genomes of Colorado (AY590688), Goose (DQ009484), PL-1 (EF199771), USA(AY579780), Human001 (AF37 -1337), GZ01 (GQ153651), VC03 (AB54 8428) and LAHA (AY640317) were 92.3%, 93.6%, 94.3%, 94.3%, 69.7%, 69.6%, 60.2 and 60.4%, respectively. Compared with the completely sequenced aMPV and hMPV, the results indicate that S-01 had a similar viral genomic structure and a consistent length of viral main protein genes with Colorado (aMPV-C^a^, AY590688) (Figure 4). The non-coding regions between the main protein genes of S-01 were similar to Goose (DQ009484) (Table 2), but the deduced amino acid sequence length of the L gene of S-01 was longer than Goose (DQ009484) (Figure 4). A high homology in the deduced amino acid sequence was found in all eight of the proteins among the other fully sequenced aMPV-C. The sequencing data placed S-01 in the C subtype of aMPV. The deduced amino acid sequence identities also show that S-01 was more homologous to hMPV than aMPV-A, aMPV-B, or aMPV-D (Table 2).

**Table 2:** Comparison of % deduced amino acid sequence identity of S-01 and other viruses

	**Size of**		**Percentage predicted amino acid identity and genome nucleotide identity (%)**^**#**^								
Name*	**nt**^**a**^	**aa**^**b**^	**aMPV-A**	**aMPV-B**	**aMPV-C**	**aMPV-D**	**hMPV**	**RSV**	**BRSV**	**MPV**	**HRSV**
N	1185	394	70.9-73.7	71.2-71.7	99.0	73.7	89.1-89.9	40.1	40.8	43.5	40.6
P	885	294	56.1-56.8	55.6-55.9	95.3-96.6	/	66.4-67.8	30.7	31.1	27.6	32.0
M	765	254	78.0	77.3-78.8	98.4-99.6	/	87.8	37.6	36.5	39.0	38.0
F	1614	537	72.3-73	72.7-72.9	98.5-99.1	/	81.6-81.8	36.5	38.2	39.8	37.1
M2-1	555	184	71.9-72.4	74.6	98.4	/	83.8-84.9	38.9	37.7	36.8	37.8
M2-2	216	71	22.2-23.6	20.8	94.4-97.2	/	56.9-58.3	19.4	22.2	8.6	22.0
SH	528	175	18.3	18.8	86.9-89.2	/	27.4-29.1	15.4	9.8	13.6	9.7
G	1758	585	14.8-15.1	15.2-15.9	60.6-82.8	17.7	21.9-25.3	15.4	12.8	7.8	18.2
L	6018	2005	62.4	62.3-62.4	95.2-96.1	/	77.6-78.2	46.0	46.3	48.7	46.1
Genome	14079	/	60.4	60.2	92.3-94.3	/	69.6-70.4	46.6	46.2	46.1	46.4

^a^Nucleotides. ^b^Amino acid; ^*^N: Nucleocapsid, P: Phosphoprotein, M: Matrix protein, F: Fusion protein, M2-1: Second matrix-1, M2-2: Second matrix-2, SH: Small hydrophobic;G: Glycoprotein, L: Large polymerase; Genome: S-01 complete genome. ^#^Genbank accession number of S01 is KF364615. The evolutionary trend of S-01 was analyzed based on nucleotide sequences of aMPV-A/FJ796703, aMPV-A/JF424833, aMPV-A/AY640137, aMPV-A/L34030, aMPV-A/DQ666911, aMPV-B/AB548428, aMPV-B/L34033, aMPV-B/JN651915, aMPV-C/AJ811991, aMPV-C/AJ811992, aMPV-C/NC0077652, aMPV-C/DQ009484, aMPV-C /AY579780, aMPV-C/AY590688, aMPV-C/EF199771, aMPV-C/EF-199772, aMPV-C/FJ977568, aMPV-D /AJ251085, hMPV/DQ843658, hMPV/EF535506, hMPV/HM197719, hMPV/AY525843, hMPV/AF371337, hMPV/AB503857, hMPV/JN184400, hMPV/GQ153651, hMPV/KC562243, hMPV/KC562233, hMPV/DQ843659, RSV/FJ614813, BRSV/AF295543, MPV/AY729016 and HRSV/JQ582843.

Our results revealed that the N, P, M, F, and L genes were highly conserved in S-01 and other metapneumoviruses. The N gene of the S-01 strain consisted of a single open reading frame (ORF) of 1185 nt, which encodes a protein of 394 aa. The length of the deduced aa of the S-01 N gene was the same as that of aMPV-C, whereas the length of the same region in aMPV-A or -B was 391 aa. The P gene contained 885 nt in a single ORF encoding a protein of 294 aa, which was 15 aa longer than that of aMPV-A and 14 aa longer than that of aMPV-B. The M gene contained 765 nt in a single ORF encoding a protein of 254 aa. All the metapneumoviruses had an M gene encoded by a single ORF of 254 aa. The F gene sequence of S-01 had 1614 nt in a single ORF encoding a protein of 537 aa. The S-01 L protein gene contained 6018 nt in a single ORF encoding a protein of 2005 aa, which had the same length as most aMPV-C, but was longer than that of the goose strain, aMPV-A, or aMPV-B (Figure 4). The deduced N, P, M, F, and L aa sequences were identical to those of aMPV-C by 99.0%, 95.3-96.6%, 98.4-99.6%, 98.5-99.1%, and 95.2-96.1%, respectively (Table 2). An analysis of the deduced five structure protein aa sequences show that S-01 was more homologous with hMPV than with aMPV-A, aMPV-B, aMPV-D, RSV, BRSV, MPV, or HRSV (Table 2).

**Figure 5:**
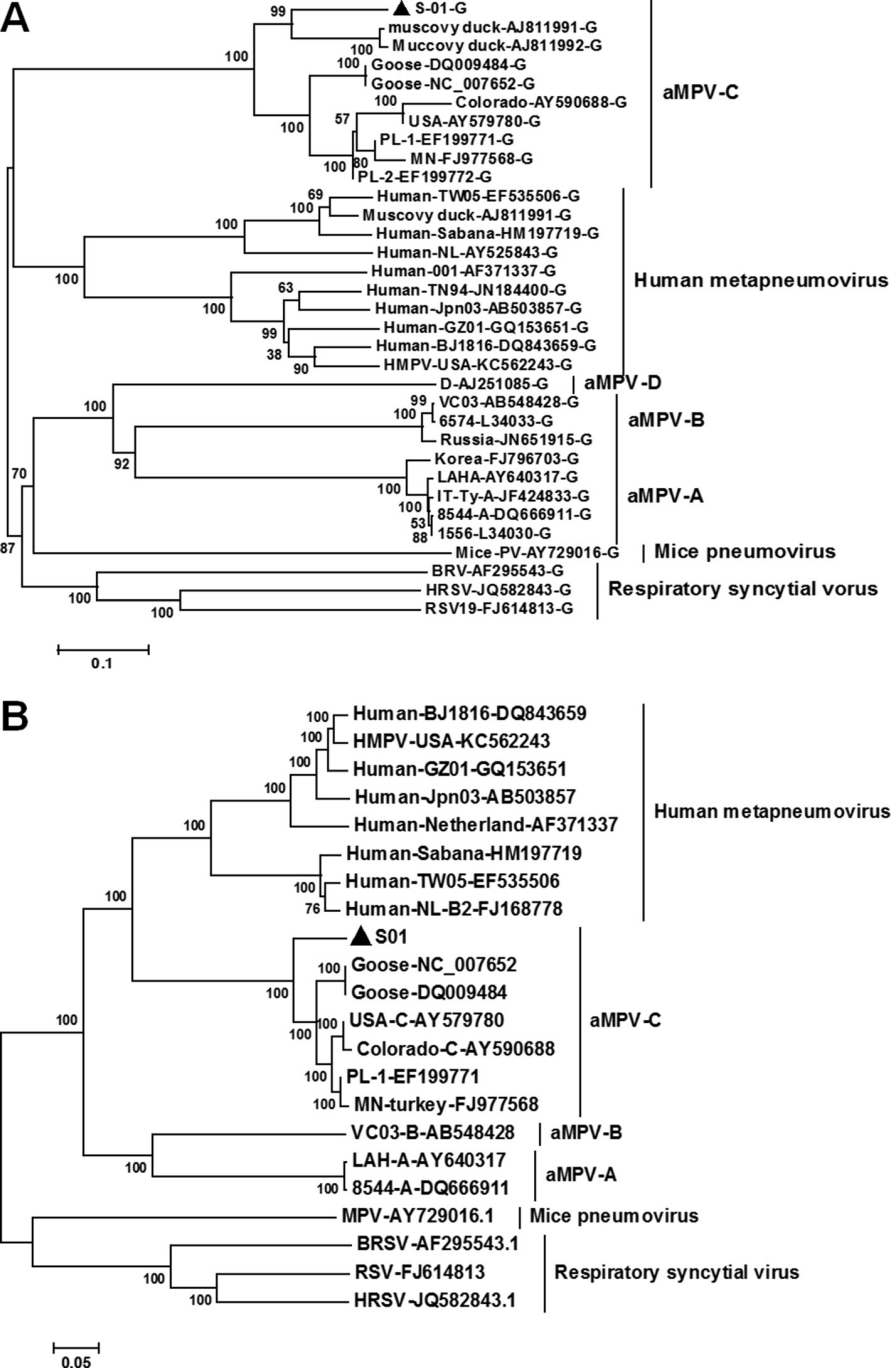
**Phylogenetic relationships of the G gene and complete genome between isolate and other pneumoviruses.** After the complete genomic sequence of S-01 was obtained, the phylogenetic tree of the G gene **(A)** and complete genome of S-01 were accomplished **(B)**, respectively.

The M2, SH and G genes had high variations in S-01 and other metapneumoviruses. The M2 gene had 765 nt encoding two ORF, M2-1 and M2-2, which were 184 aa and 71 aa long in S-01, respectively (Figure 4). The deduced M2-1 aa sequence in S01 was more conserved (71.9–98.4%) compared to M2-2 (20.8–97.2%) in the other metapneumoviruses (Table 2). The SH gene of S-01 consisted of 528 nt encoding one ORF of 175 aa. The deduced aa sequence analysis revealed that SH shared an 86.9-89.2% identity with the SH protein of aMPV-C, and only 9.7-29.1% identity with the SH protein of other metapneumoviruses (Table 2). The G gene had the highest variation between S-01 and other metapneumoviruses. The S-01 G gene contained 1758 nt in a single ORF encoding a protein of 585 aa, which had the same length as most aMPV-C, but was longer than that of some aMPV-C strains (such as PL-1, PL-2, and AY579780 strain) and other metapneumoviruses (Figure 4). The deduced aa identity of S-01 was 60.6%, 79.9%, 79.9%, 67.4%, 82.8%, 82.4%, 77.0%, 77.4%, 77.1% with that of aMPV-C/USA/Colorado/AY590688, aMPV-C/Canada/Goose/DQ009484, aMPV-C/Canada/Goose/NC0077652, aMPV-C/USA/MN/FJ977568, aMPV-C/France/duck/AJ81-1991, aMPV-C/France/duck/AJ811992, aMPV-C/Korea/PL-1/EF199771, aMPV-C/Korea/PL-2/EF-199772 and aMPV-C/USA/ AY579780, respectively, but was lower with other metapneumoviruses (<25.3%) (Table 2). Importantly, the phylogenetic analysis of the G gene shows that S-01 was the most closely related to strains isolated in Muscovy ducks in France, aMPV-C/France/duck/AJ811991 & AJ811992 (Figure 5A).

The phylogenetic analysis of the complete genome indicates that S-01 belonged to the aMPV-C subgroup and was closely related to hMPV (Figure 5B). These results demonstrate that the S-01 virus, isolated from Chinese Muscovy ducks, was most closely related to hMPV and belonged to the aMPV-C family.

## Discussion

Muscovy ducks were introduced from Europe to China over 400 years ago. To date, no data have shown that Muscovy ducks were infected by the aMPV-C virus strain in China. Our research is the first report showing that subtype C aMPV was responsible for the epidemic occurring in Muscovy ducks in China. The only case of aMPV-C isolation from Muscovy ducks was reported in France in 1999 [17]. Our result shows that the G protein of S-01 shared approximately 82% identity with that of French isolates, however, it seems extremely low as an ancestor of S-01. aMPV-C has been demonstrated in wild geese in Canada [30]. Although there has been no report about isolation of aMPV-C from wild birds in China, aMPV-C infection of Chinese wild ducks cannot be ruled out and remains to be determined. How the aMPV-C was transmitted into Muscovy ducks in China, and whether the virus outbreak lineage has, or will become enzootic in China requires further investigation.

The identification of a virus depends on the characterization of the gene structure and homology analysis [14]. Seal [30] initially confirmed the new subgroup C aMPV through aMPV-CO viral gene structure and phylogenetic analyses of the M and F proteins, together with the serological uniqueness of the US APV isolates. Bayon-Auboyer et al. identified aMPV-D by phylogenetic analysis of the F, G and L genes in isolates Fr/85/1 and Fr/85/2 [31]. S-01 had comparable similarity to the viral structure with metapneumovirus, and shared high homology of deduced amino acid identities in all eight proteins with other fully sequenced aMPV-C. Our results strongly indicate that the new isolate in China belonged to the C subtype of aMPV.

The four subgroups of aMPV were distributed unevenly, perhaps due to regional and species-specific differences. Subgroups A and B were found worldwide in chickens or turkeys [4],[32]–[35]. Subgroup D has only been isolated in France [31], and subgroup C has been found in Europe (Muscovy duck) [15], North America (turkey) [6], and South Korea (pheasant) [7]. Subgroup C is related to a more extensive host range than the other subgroups [6],[7],[15],[17],[36],[37]. Although subgroup C aMPV was reported in South Korea [7], our research is the first to report that subgroup C was initially isolated from Muscovy ducks in Asia. Experiments are now under way to determine the presence of aMPV-C in other species of birds in South China (Sun and Li, unpublished data).

The recent aMPV-C epidemic, which has been circulating in southern China for about two years, was more complex and worse than previous outbreaks, such as the outbreak occurring in France [17]. Once the disease of aMPV-C with the egg-drop symptom occurred in a laying flock, all birds in the rest of the flocks on the same farm would exhibit similar symptoms rapidly, indicating that the virus had strong transmission ability. The mortality rate shows a huge range across different time periods, which was more than 10% during 2010 to 2011, and was rare after 2012 (data not shown). Van de Zande et al. demonstrated that aMPV might act as a primary agent that predisposes infected ducks to *E. coli* colonization and invasion [38]. *Pasteurella* anatipestifer was frequently isolated from the diseased ducks in the initial outbreak as the second pathogen, but *E. coli* infections were more common after 2012 (data not shown). The varying mortality rates may be related to the different main secondary pathogens isolated from the two periods. In addition, the therapeutic effects of vaccines and antibiotics might also play a role in the different mortality rates.

Isolating aMPV from field samples is not easy [14]. Kwon et al. reported that two aMPV-As out of the 25 detected aMPV were successfully isolated in Vero cells [29]. Our study had similar results. For reasons that were not apparent, the isolation of subgroup C virus was more difficult than that of subgroups A or B [6]. This might correspond to the fact that subgroup C does not cause ciliostasis [6],[39]. Subgroup C aMPV from duck, turkey, geese and sparrow was isolated by initially inoculating SPF chicken embryos for several passages, and then inoculating them onto cell lines such as Vero cells, or inoculating them onto SPF-chicken or turkey embryo fibroblasts until CPE was evident [6],[17],[40]. We previously used similar methods as described above, but it did not work. This study is the first to use duck embryos as the primary means of isolation for subgroup C aMPV.

aMPV-C can be divided into two sublineages, aMPV-C^a^ and aMPV-C^b^, according to the different lengths of the G protein (Figure 4). Interestingly, PL-1 and PL-2, which were isolated from pheasants in South Korea and identified as aMPV-C^a^, shared similar length of the G protein with USA/AY579780 and high identity (87.9-97%) with North-American isolates, including aMPV-C^b^ (Canada/Goose/DQ009484, USA/Colorado/AY590688, USA/MN/FJ977568 and Canada/Goose/NC0077 -652). S-01 resembled French isolates (France/duck/AJ811991&AJ811992) the most and had higher identity with them than with North-American isolates. North-American and French isolates of aMPV-C belonged to significantly different genetic lineages [15]. This may suggest that S-01 and PL-1 or PL-2 belong to distinct sub-lineages, indicating that the situation of aMPV-C infection in Asia is complex and the distribution of the virus is highly diverse. The relationship between S-01 and PL-1 needs more surveillance studies.

The sequence analysis revealed that the G gene sequence of aMPV-C^a^, aMPV-A, aMPV-B, aMPV-D and hMPV was shorter than that of aMPV-C^b^. All of the deletion regions may be related to host specificity, but more experimental evidence is needed to confirm this hypothesis. It was estimated that cross-species transmission of metapneumoviruses from birds to humans occurred approximately 200 years ago [41]. At the genetic and antigenic levels, it is plausible that an aMPV-C-like virus was the ancestor of hMPV [41]. Our results support the findings that aMPV-C^b^ may be the ancestor of metapneumoviruses.

Prior to our study, there was no published report that successfully replicated the disease with experimental aMPV-C infection in Muscovy ducks. Although Toquin et al. effectively isolated aMPV-C from Muscovy ducks, they did not mention to reproduce a clinical case of aMPV-C infection in Muscovy ducks [17]. We initially encountered a similar problem until we adjusted cell-passage strain to duck embryo-passage strain. An attenuated vaccine against subgroup C could be developed via serial propagation of virulent aMPV-C in Vero cells [42]. The S-01 virus might have reduced virulence by passage in Vero cells. The G protein is the viral attachment protein known to be an important immune response antigen and virulence factor, and this protein has variance in all metapneumoviruses [43]. The G gene-deduced aa identity between Chinese and European aMPV-C isolates was only approximately 82%. A low identity of G protein may be related to the difference in virulence for Muscovy ducks. We speculate that S-01 may have higher virulence than the French isolates. Various stress conditions, including temperature, artificial insemination, and ambient air quality, might also play an important role in aMPV-C infection.

Hoogen et al. published the genomic sequence of hMPV (hMPV-001 strain), allowing for detailed comparison of aMPV and hMPV [45]. Comparisons of the N, P, M, F, and M2 proteins indicate that the highest aa identity (overall 80%) was between aMPV-C and hMPV, and it was significantly higher than the aa identity between hMPV and aMPV-A or aMPV-B [44]. Our work had similar findings. In addition, S-01 binded to the antibody of the F protein of the hMPV (Figure 3D). The hMPV have been present in humans for more than 60 years. [45]. It has been hypothesized that either of the two viruses can cross-infect each other, i.e., hMPV might infect turkeys and aMPV-C might infect humans [46]. It should be noted that S-01 was isolated from Guangdong, China, where hMPV were present many years ago [47]. Further studies are needed to determine the relationship between aMPV-C and hMPV.

Ducks seem to be susceptible carriers for many zoonotic viruses, such as avian influenza virus and BYD virus, and they play an important role in the transmission and evolution of these viruses [48]. Ducks were suspected to play a role as nonclinical carriers of aMPV [49]. Because duck consumption is particularly important in China, effective control of aMPV-C infections in duck populations should be considered. Future worldwide surveillance should also be enhanced.

## Competing interests

The authors declare that they have no competing interests.

## Authors’ contributions

SKS, FC and HYL conceived and designed the study. SKS, SC, WL, JJL, GWL, SYF, CL generated the raw data. SKS, JPL and JPQ analyzed raw data. SKS performed statistical analysis. SKS wrote paper. HYL revised the paper. All authors read and approved the manuscript.
